# Biological assessments of multifunctional hydrogel-decorated implantable neural cuff electrode for clinical neurology application

**DOI:** 10.1038/s41598-017-15551-x

**Published:** 2017-11-10

**Authors:** Han-Jun Kim, Dong Nyoung Heo, Yi Jae Lee, Sang Jin Lee, Ji Yoon Kang, Soo Hyun Lee, II Keun Kwon, Sun Hee Do

**Affiliations:** 1 Konkuk University, Department of Clinical Pathology, College of Veterinary Medicine, Seoul, 05029 Republic of Korea; 2 Kyung Hee University, Department of Dental Materials, School of Dentistry, Seoul, 02477 Republic of Korea; 3 Korea Institute of Science and Technology, Center for BioMicrosystems, Seoul, 02792 Republic of Korea

## Abstract

The implantable cuff electrode is an effective neuroprosthetic device in current nerve tissue engineering. However, biocompatibility and stability are still a serious dispute in terms of *in vivo* function and continuous monitoring. In this regard, assessing the host’s biological response to biomaterials is one of the key factors of chronic implantation. In this article, we analyzed the peripheral nerve specific-biological responses to the application of multi-functional hydrogel-coated electrodes. The surface of the cuff electrode was modified using a multifunctional hydrogel composed of PEG hydrogel, cyclosporin A(CsA)-microsphere(MS) and electrodeposited PEDOT:PSS. Through our approach, we have found that the multifunctional hydrogel coatings improve the neural electrode function, such as peak-to-peak amplitude increase. Additionally, the multifunctional hydrogel coated electrodes exhibited improved biocompatibility, such as reduced apoptotic properties and increased axonal myelination. Furthermore, 12 genes (*BDNF*, *Gfra1*, *IL-6*, *Sox* 10, *S100B*, *P75* ^*NTR*^, *GAP43*, *MBP*, *MPZ*, *NrCAM*, *NE-FL*, *CB1*) were upregulated at 5 weeks post-implant. Finally, double immunofluorescence revealed the effect of endocannabinoid system on neuroprotective properties and tissue remodeling of peripheral nerves during cuff electrode implantation. These results clearly confirmed that multifunctional hydrogel coatings could improve electrode function and biocompatibility by enhancing neuroprotective properties, which may provide a valuable paradigm for clinical neurology application.

## Introduction

Neural prosthetics are promising tool for the rehabilitation of patients with disabled or weakened neuromuscular functions due to nervous system injuries. Regardless of the types of injury, a part of the nerve could be left intact and used as a source of functional restoration. For this purpose, the cuff electrode as means of neural prosthetic has been significantly employed during several decades. The cuff electrode is an extra-neural electrode placed around the nerve to exchange control signals between the central nervous system and the extremities or organ^1,2^. Among the several types of electrodes, the cuff electrode has diverse advantages such as reduced mechanical stress, non-invasiveness (relatively), and a high stability over time^3–5^. Using these merits, the nerve electrode has been well applied to aid in foot drop correction^6^, arm and hand function^7,8^, respiratory output monitoring^9^, and bladder constriction^10^.

Despite the advantages and efficacy of cuff electrodes, there is still a unexplained limitations such as failure of long-term implantation. Assessing the host’s biological response to the biomaterial is one of the most key factors in successful *in vivo* function and ongoing monitoring. The host’s biological responses to foreign materials, e.g. cuff electrodes, are regulated in a complex manner, and the response lasts up to the electrode placed around the nerve^11,12^. The host’s biological responses begin with the surface biofilm coating and progresses to acute inflammation, foreign body response. In the final stage of the foreign body reaction, this reaction eventually leads to target tissue remodeling^11,12^. Previous studies have reported that cuff electrode implantation could alter the DNA content of the target tissue^13^. In particular, studies have shown that, in the case of peripheral nerves, gene expression levels such as TNF-α and TGF-β1 could be up-regulated by cuff electrodes^14^. For this reason, it is essential to understand the tissue-specific mechanisms of successful/unsuccessful implantation by analyzing biological events at the implant-tissue interface.

The endocannabinoid system (ECS) is distributed in the nervous system and could be modulated in response to extrinsic stress by altering the expression of cannabinoid receptor (CB) 1 and 2^15,16^. Several studies have shown that CB1 is expressed in not only in the dorsal root ganglion (DRG) neurons but also in both large-diameter non-nociceptive and small-diameter nociceptive neurons^17,18^. In addition, activation of cannabinoids, CB1 or CB2 are closely linked to adult neurogenesis such as differentiation and myelination^19^. In this regard, assessing the levels of expression of ECS in neural tissues during neural cuff transplantation is worth studying as a biomarker of neural tissue.

In view of the importance of the above studies, biological aspects as *in vitro* and *in vivo* assessments should be integral especially for clinical application. In this article, we mainly focoused on the biological assessment of the multifunctional hydrogel-decorated implantable neural cuff electrode to confirm functionality and biocompatibility. In the most previous our report, Heo *et al*. devised that the surface-functionalized cuff electrode which was coated with polyethylene glycol (PEG) hydrogel, cyclosporine A (CsA) loaded microsphere, and electrodeposited poly(3,4-ethylenedioxythiophene) polystyrene sulfonate (PEDOT:PSS)^20^. From this approach, the functional cuff electrode yielded not only the enhanced electrochemical effectiveness but also reduced inflammatory responses. As advanced research based on our previous report in this study, we analyzed biofunctionality such as electroneurograms (ENG) analysis from the experimental group at 2 weeks and 5 weeks. Additionally, biocompatibility effects of the multi-functional hydrogel coating were evaluated using histo-morphological and histochemical staining. Furthermore, the real-time PCR and double immunofluorescence staining were performed to reveal the mechanisms underlying biocompatibility. Finally, we estimated the association of endocannabinoid system in enhanced neuroprotective properties of multi-functional hydrogel coated cuff electrodes for *in vivo* neural interfacing.

## Results

### Fabrication of the multifunctional hydrogel-coated electrodes and electroneurogram

**Figure 1:**
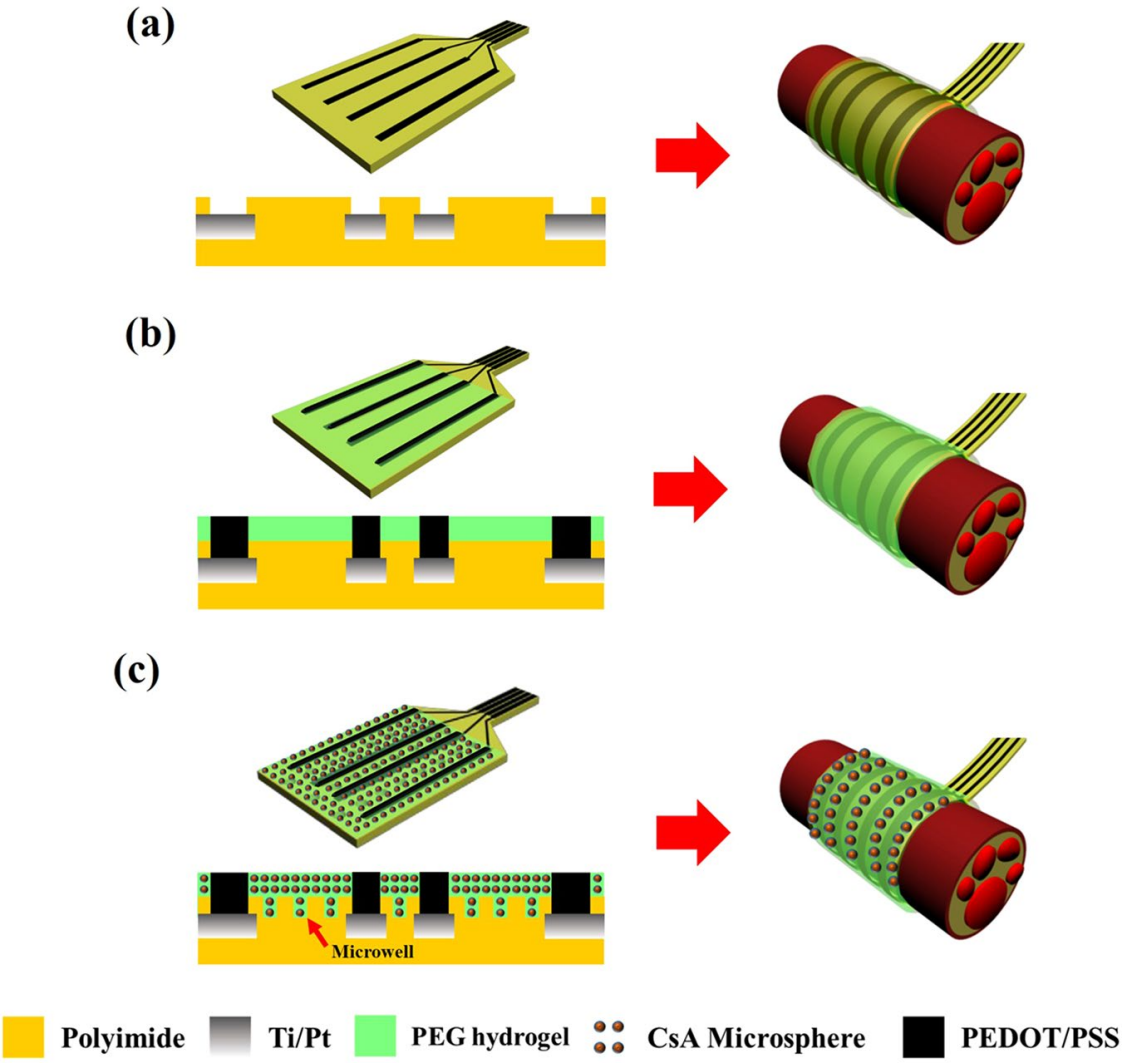
Schematic illustration of the fabrication and cross-sectional views of the multi-functional hydrogel coated recording electrode. (**a**) Polyimide Pt electrode (Con). (**b**) PEDOT:PSS electrode with PEG hydrogel (HP) and (**c**) microwell-type PEDOT:PSS electrode with CsA loaded microsphere-PEG hydrogels (HMP).

The fabrication process of the multifunctional hydrogel-coated functional nerve cuff electrode is shown in Fig. 1. The process used to fabricate the nerve cuff electrode was described in detail in our previous reports^20^. Three kinds of electrodes were prepared and analyzed for the experiments (Fig. 1):

1. Bare cuff electrode (Con)
2. Polyethylene glycol (PEG) hydrogel and PEDOT:PSS coated cuff electrode (HP)
3. Microwell-patterned cuff electrode coated with cyclosporine A loaded microsphere (MS) containing PEG hydrogel and PEDOT:PSS (HMP)

As shown in Fig. 1a, the functional cuff electrodes consisted of a four-line electrode of platinum (Pt) deposited sites on a polyimide (PI)-based background. The electrode had a rectangular shape and was 4 mm in length and 0.25 mm in width. An additional surface modification process was performed in the HMP group such that the microwell patterned electrode was made. This process increased the surface area at which high doses of the CsA could be loaded and increased physiological adhesion properties. The PEG and CsA loaded microsphere composite hydrogels were mixed and coated on the surface-modified PI-based cuff electrode (Fig. 1b,c). Finally, the PEDOT:PSS polymer was electro-polymerized on four Pt line electrode sites in HP and HMP groups.

**Figure 2:**
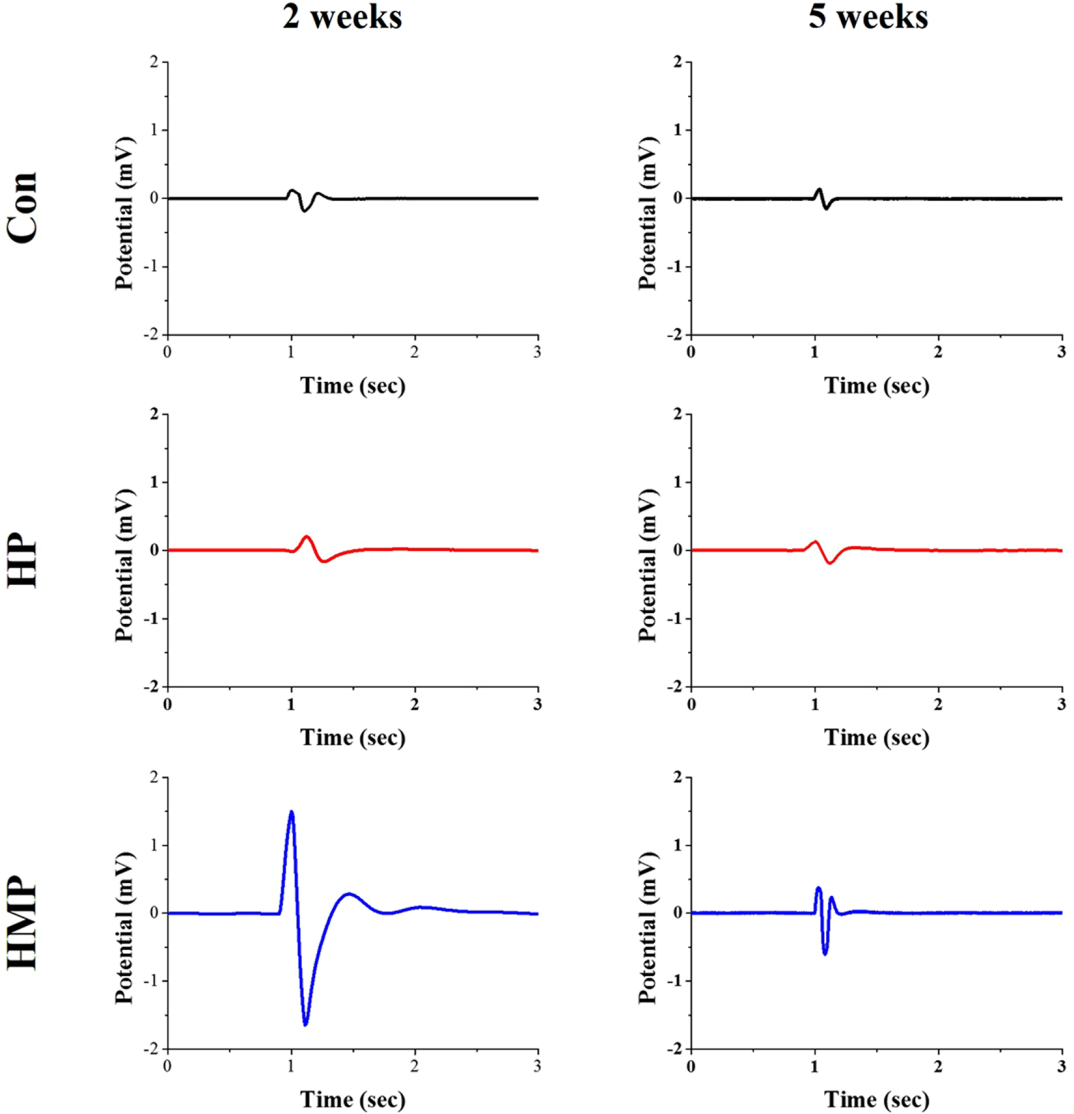
Comparison of *in vivo* sciatic nerve signal recordings to the electrical stimulation at 2 weeks and 5 weeks post-implantation. An electrical current was delivered through the stimulation electrode using a biphasic current pulse with 300 μA peak current and 100 μs duration per phase.

Figure 2 shows the stimulus artifacts and the electrically evoked ENG signals of the Con, HP, and HMP groups which were recorded using surface-modified electrodes. Single biphasic electrical pulse stimulation was applied by the co-implanted stimulation cuff electrode (non-coated bare electrode). In the 2 weeks post-implant, the evoked ENG peak-to-peak signal was observed in the surface-modified groups (HP and HMP group), but not in the Con group. The amplitude of the peak-to-peak signals of HP group (0.366 mV) were slightly higher than Con group (0.306 mV). HMP group showed significantly increased peak-to-peak signals than other groups (3.145 mV). While the evoked ENG signal of the Con group almost disappeared at 5 weeks, the HMP groups still exhibited the action potentials. The amplitude of the peak-to-peak signals was also higher in the HMP group (0.989 mV) than in the Con (0.289 mV) and HP (0.314 mV) group. Taken together, HMP group exhibited relatively constantly enhanced ENG recording properties than the other groups including clearly distinguishable ENG peak potential and higher peak-to-peak signal amplitude.

### Histological and histochemical analysis

**Figure 3:**
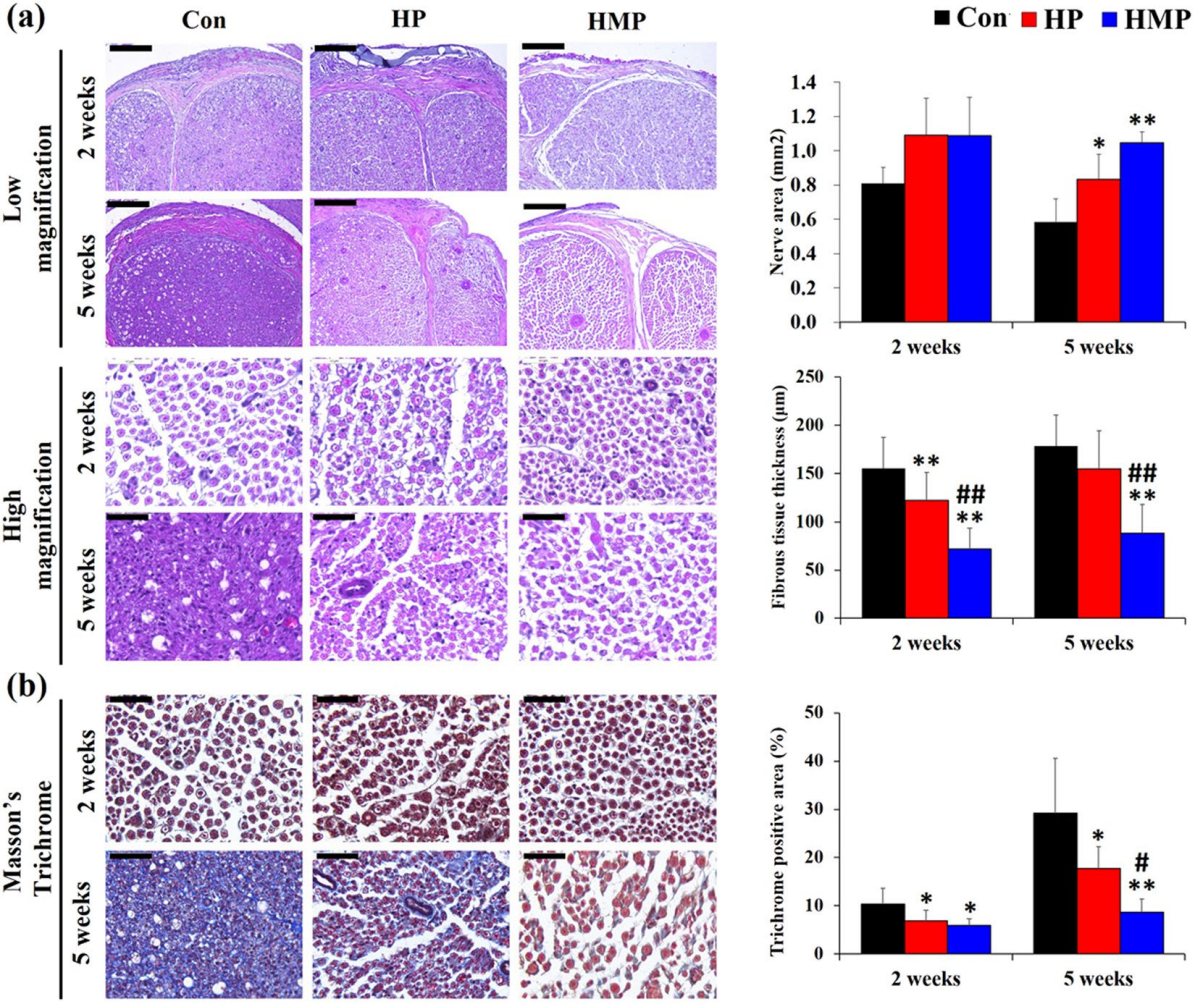
H&E and Masson’s trichrome stain of the sciatic nerve at 2 and 5 weeks post-implantation. Representative micrographic images and statistical analysis of staining with (**a**) H&E and (**b**) Masson’s trichrome staining. Scale bar; low magnification (200 μm), high magnification (50 μm), Masson’s trichrome (50 μm). Data were expressed as mean ± SD, three samples for each group and each time point. *Compared to Con; ^#^Compared to HP; ^*, #^ *p* < 0.05, ^**, ##^ *p* < 0.01; One-Way ANOVA analysis.

Figure 3a shows the HE staining of the sciatic nerves implanted with the Con, HP, and HMP electrodes at 2 weeks and 5 weeks post-implantation. The cross-sections of the nerve exhibited epineural fibrosis and a mild degree of endoneurial degeneration in the Con and HP groups at 2 weeks. The Con group showed a marked degree of endoneurial fibrosis at 5 weeks that the margin of the axons was not distinct. However, degenerative features such as axonal integrity loss and fibrous tissue infiltration were minimal in the HMP group.

Quantitative data showed that the Con group had a significantly decreased nerve area (mm^2^) at 5 weeks than the HP and HMP group (*p* < 0.05 for HP, *p* < 0.01 for HMP). In addition, the thickness of epineural fibrous tissue decreased in the HP (21.3%) and HMP (53.5%) groups at 2 weeks (*p* < 0.01) as compared with the Con group. However, the HP group showed increased epineural fibrous tissue infiltration at the end point of the experiment and at that point, the difference between the Con and HP groups was not statistically significant. Only the HMP group had a consistently decreased epineural fibrous tissue thickness (50.6%, *p* < 0.01) compared to the Con group at 5 weeks.

Masson’s trichrome staining was performed in the experimental groups to determine whether surface modification affects the degree of endoneurial fibrosis (Fig. 3b). The experimental groups showed no marked distinct endoneurial fibrosis for up to 2 weeks. However, after 5 weeks, massive endoneurial fibrosis was observed in the Con group, and most of the axons were surrounded by the trichrome-positive areas. Quantitative analysis revealed that the surface modification groups (HP, HMP) had significantly less endoneurial fibrosis than the Con group throughout the experimental period (HP vs. Con, 5 weeks; 39.5%/HMP vs. Con, 5 weeks; 70.4%, *p* < 0.05). However, at the end point of the experiment, endoneurial fibrosis progressively increased not only in the Con group but also in the HP group. Only the HMP group showed a relatively constant reduced endoneurial collagenous content (*p* < 0.05).

**Figure 4:**
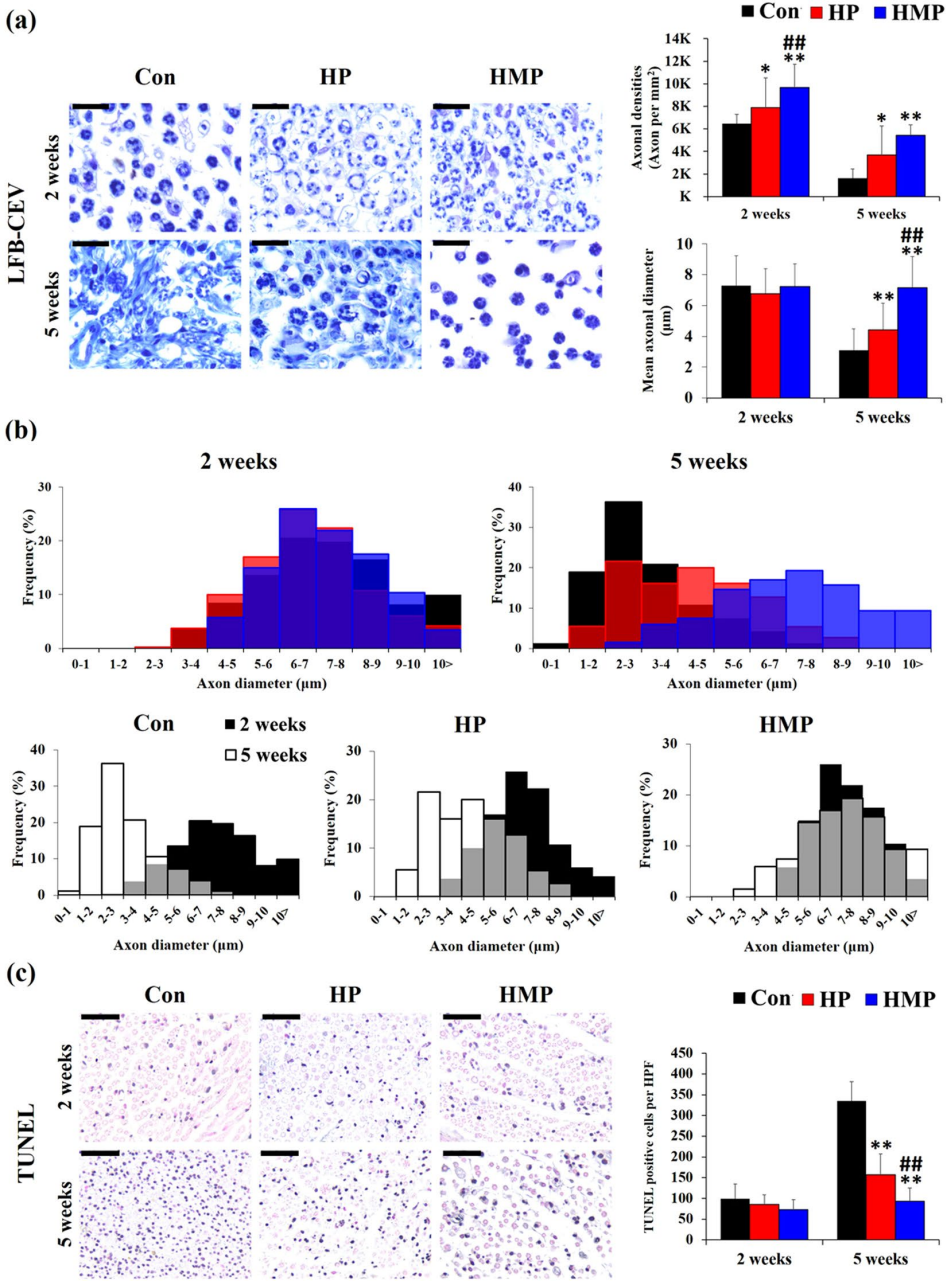
LFB-CEV and TUNEL staining of the sciatic nerves in the experimental groups at 2 and 5 weeks post-implantation. (**a**) Representative images and statistical analysis of Luxol fast blue-cresyl echt violet (LFB-CEV) staining. Scale bar = 20 μm, (**b**) Axon diameters distribution in the experimental groups (LFB-CEV staining). (**c**) Representative images and statistical analysis of TUNEL staining. Scale bar = 50 μm. Data were expressed as mean ± SD, three samples for each group and each time point. ^*^Compared to Con; ^#^Compared to HP; ^*, #^ *p* < 0.05, ^**, ##^ *p* < 0.01; One-Way ANOVA analysis.

Further, axonal structural maintenance was analyzed by LFB-CEV (Fig. 4a,–b) and TUNEL staining (Fig. 4c). When the peripheral nerve damage progresses chronically, the nerve tissue could undergo neural tissue remodeling and degeneration such as endoneurial fibrosis, myelin detachment/degeneration, and axonal cell death^21,22^.

The myelin content in the experimental groups was analyzed using LFB-CEV staining (Fig. 4a). The Con group showed an increase in the numbers of small sized LFB-CEV positive-myelinated axons at 5 weeks after implantation compared to 2 weeks. In the HMP group, there was a relatively large axon at 5 weeks, and individual LFB-CEV positive myelinated axons were located in the endoneurial area without surrounding fibrous tissue infiltration. Quantitative analysis of axonal densities (LFB-CEV positive axons per mm^2^) showed that the HP and HMP group had significantly higher axonal densities than the Con (Con vs. HP, 2 weeks; 122%, 5 weeks; 224%, Con vs HMP, 2 weeks; 149%, 5 weeks; 332%, /HP vs. HMP, 2 weeks; 122%, *p* < 0.01). In addition, the mean axon diameter of HMP was significantly larger than those of Con and HP groups at 5 weeks (Con vs. HMP; 233%, HP vs. HMP; 162%, *p* < 0.01). During the experimental periods, the distribution of axon diameters was biased toward the small axons in Con and HP group (Fig. 4b, Kolmogorov-Smirnov test, *p* < 0.01). This small axon diameter tendency (axonal atrophy) was more pronounced in the Con group than in the other groups. Only the HMP group showed a relatively constant axon diameter distribution patterns and showed a tendency to be significantly larger than Con and HP (Con, HP vs. HMP; *p* < 0.01).

TUNEL staining was used to assess apoptotic cell death in the experimental groups (Fig. 4c). TUNEL positive cells were sporadically distributed in endoneurium in all the groups at 2 weeks which was not statistically different between the experimental groups. However, the number of TUNEL-positive cells in endoneurium was significantly higher in the Con and HP groups during the experimental period. Despite the constant increase in apoptotic cells, the HP group showed significantly less apoptotic cells (53.1%, *p* < 0.01) at 5 weeks than the Con group. Similar to the results of the above endoneurial fibrosis and myelin remnants, only HMP group showed consistently low apoptotic cell count at 5 weeks (Con vs. HMP; 62.2%, *p* < 0.01). HMP group showed less apoptotic cells (59.3%, *p* < 0.01) at 5 weeks than HP group. Taken together, surface modification through PEG hydrogel coatings containing CsA microsphere produced reduced interface fibrous tissue infiltration, maintenance of axonal myelin content and axonal cell survival during chronic implantation.

### Gene expression analysis

**Figure 5:**
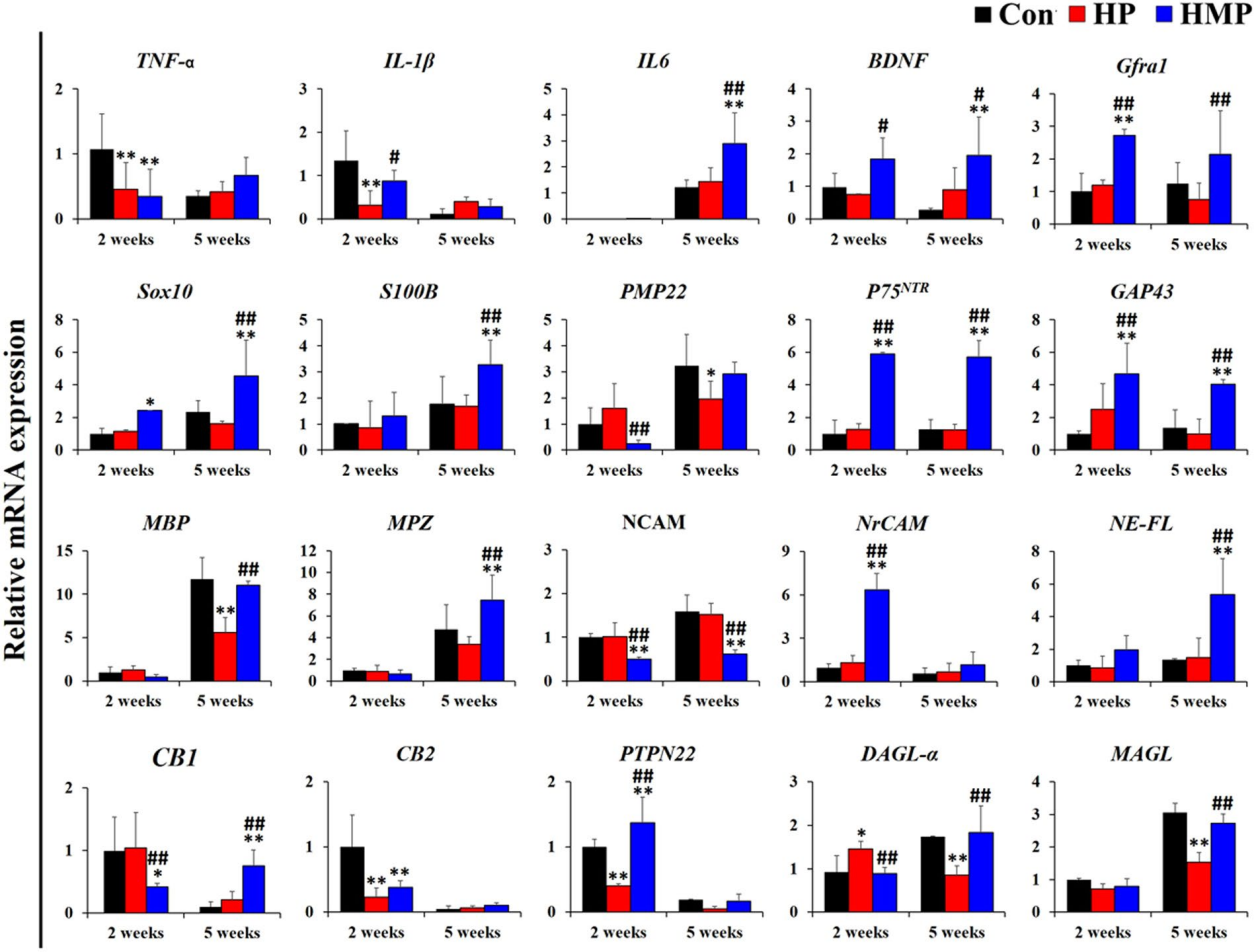
Analysis of gene expression in the experimental groups at 2 and 5 weeks post-implantation. Real-time PCR was used to analyze mRNA expression level in the experimental group (Con – Black, HP – Red, HMP – Blue) (n = 3 per each group and each time point). Primers were categorized as neuroinflammation-, neurotrophic factors-, Schwann cell phenotype-, myelination-, adhesion and axonal cytoskeleton- and the endocannabinoid system. Results were expressed as mean ± SD from triplicate of experiments. ^*^Compared to Con; ^#^Compared to HP; ^*, #^ *p* < 0.05, ^**, ##^ *p* < 0.01; One-Way ANOVA analysis.

To investigate the host’s biological response to the implantation of surface-modified electrodes, quantitative real-time PCR analysis was performed as shown in Fig. 5. Relative gene expression values were obtained by using the 2−ΔΔCt method (ΔCt sample−ΔCt calibrator) with the mean of the 2-week Con group value was used as a calibrator. Primers are listed in in Table 1 as neuroinflammation, neurotrophic factors, Schwann cell phenotype, myelination, adhesion, and axonal cytoskeleton and endocannabinoid system.

**Table 1:** Primer sequences for Real-time PCR.

Neuroinflammation	*TNF-a*	CCCCGACTATGTGCTCCTCAC	AGGGCTCTTGATGGCGGA
	*IL-1β*	CCTCAAGGGGAAGAATCTAT	GAGGTGCTGATGTACCAGTT
	*IL-6*	GGGACTGATGTTGTTGACAG	TGTTCTTCACAAACTCCAGG
Neurotrophic factor	*BDNF*	CAAAAGGCCAACTGAAGC	CGCCAGCCAATTCTCTTT
	*Gfra1*	CAGGCAGTTCTTCGACAAGGTT	TGGGCCTCTCTCGTTCTTCA
Schwann cell & Myelination	*Sox10*	CGAATTGGGCAAGGTCAAGA	CACCGGGAACTTGTCATCGT
	*S100B*	TGCCCTCATTGATGTCTTCCA	GAGAGAGCTCGTTGTTGATGAGCT
	*PMP22*	AATAATCCGCTGCCCGAATCAATG	CTCCGCCTTCAGGGTCAAGTG
	*p*_75_^*NTR*^	CCAGCAGACCCATACGCAGAC	GCCAGATGTCGCCAGGTATCC
	*GAP43*	CTTTCCTTAGGTTTGGCTTCA	TAAACAAGCCGATGTGCCT
	*MBP*	GCACAGAGACACGGGCATCC	CGGGCATGAGAAGGCAGAGG
	*MPZ*	CTGTTGCTGCTGTTGCTCTTCTAC	TTGGTGCTTCGGCTGTGGTC
Adhesion & Neuroskeletal	*NCAM*	GCAGGTAGATATTGTTCCCA	GGTTTGGGCTCAGCTTCTCC
	*NrCAM*	AAAGGGAAACCTCCCCCAAG	TGTTGATGACAAGGGTTCCTGA
	*NE-FL*	TCTGCGTACTCCAGCTACTC	AGCTGTGCCTTCTCCTGTGT
Endocannabinoid system	*CB 1*	CTACTGGTGCTGTGTGTCATC	GCTGTCTTTACGGTGGAATAC
	*CB 2*	GAGGCTGAGACTCTGGTCCTGAA	CTGGGCAATGGGGACGGACG
	*PTPN22*	TGGTCGTGGGAGAGCCGCTT	GGGCCACTTTTTGCGCCTGC
	*DAGL-a*	GGCCGCACCTTCGTCAAGCT	ATCCAGCACCGCATTGCGCT
	*MAGL*	CATGGAGCTGGGGAACACTG	GGAGATGGCACCGCCCATGGAG
Reference gene	*18 s rRNA*	ACGGACCAGAGCGAAAGCAT	TGTCAATCCTGTCCGTGTCC
	*HPRT1*	GCCCCAAAATGGTTAAGGTTGCAAG	ATCCAACAAAGTCTGGCCTGTATCC
	*β actin*	AGGCCAACCGTGAAAAGATG	ACCAGAGGCATACAGGGACAA
	*GAPDH*	AACTCCCTCAAGATTGTCAGCAA	GGCTAAGCAGTTGGTGGTGC

The mRNA expressions of pro-inflammatory cytokines such as *TNF-α* (tumor necrosis factor-alpha) and *IL-1β* (interleukin-1beta) were significantly downregulated in the HP and HMP groups than in the Con group at 2 weeks (*p* < 0.01). However, the level of *IL-6* expression was significantly increased in the HMP group at 5 weeks (*p* < 0.01). The expression levels of *BDNF* (brain-derived neurotrophic factor) and *Gfra1* (glial cell line derived neurotrophic factor family receptor alpha 1), which are related to neurotrophic factors, were consistently upregulated in the HMP group during the experiment. However, the *BDNF* and *Gfra1* gene expression levels in the Con and HP groups did not change significantly during the course of the experiment.

Expression levels of Schwann cell phenotype-related genes such as transcription factor *Sox 10* (SRY-box 10), *S100B*, *PMP22* (peripheral myelin protein 22), *P75* ^*NTR*^ (p75 neurotrophin receptor), and *GAP43* (growth associated protein 43) were also different between the experimental groups. The expression levels of *S100B*, *P75* ^*NTR*^, and *GAP43* in the Con and HP groups did not change significantly during the experiment. On the other hand, mRNA expression levels of *Sox10* and *S100B* were significantly higher in the HMP group (Con, HP vs. HMP; *p* < 0.01). Furthermore, *P75* ^*NTR*^ and *GAP43* were consistently upregulated in the HMP group during the experiment (Con, HP vs. HMP; *p* < 0.01). During the experimental period, expression levels of *MBP* (myelin basic protein) and *MPZ* (myelin protein zero) were also increased in the HMP group (*MPZ*: Con vs. HMP, *p* < 0.01, *MBP*, MPZ: HP vs. HMP, *p* < 0.01). Expression of adhesion molecules and axonal cytoskeletal genes in the experimental groups was found to be altered in relation to upregulation of myelination-related genes. The expression level of *NCAM* (neural cell adhesion molecule) was relatively constant and decreased in HMP through experiments (Con, HP vs. HMP; *p* < 0.01). However, the expression levels or *NrCAM* (neuronal cell adhesion molecule) and *NE-FL* (neurofilament light chain) were higher in the HMP group than in the Con and HP groups at 2 and 5 weeks (Con, HP vs. HMP: *p* < 0.01).

It is well known that the endocannabinoid system (ECS) is expressed throughout the nervous system and could be regulated in response to extrinsic stress by altering the expression of CB 1 and 2^15,16^. We analyzed the association of ECS-related mRNA expression in axonal maintenance and regeneration in the experimental groups. The expression levels of *CB1* (cannabinoid receptor 1) in the Con and HP groups were higher than the HMP group at 2 weeks (Con vs. HMP: *p* < 0.05, HP vs. HMP: *p* < 0.01). However, the *CB1* mRNA expression level in the HMP group gradually increased during the experiment (Con, HP vs. HMP: *p* < 0.01). In particular, it was found that *CB1* expression of the HMP group was significantly increased at 5 weeks, similar to changes in the expression of the neurotrophic factors (*BDNF*, *Gfra1*), axonal maintenance and Schwann cell activation (*Sox10*, *S100B*, *PMP22*, *P75* ^*NTR*^, *GAP43*), and myelination (*MBP*, *MPZ*, *NE-FL*) related genes. On the other hands, the *CB2* (cannabinoid receptor 2) expression levels at 2 weeks in the HP and HMP groups were lower than that of the Con group (*p* < 0.01). In our results, the expression pattern of *CB2* was low in the surface-modified groups (HP, HMP) at 2 weeks, similar to the expression pattern *TNF-α* and *IL-1β* mRNA.

We also analyzed the gene expression levels of biosynthesis and degradation enzymes of endogenous cannabinoids such as *PTPN22* (protein tyrosine phosphatase non-receptor type 22), *DAGL-α* (diacylglycerol lipase alpha), and *MAGL* (monoacylglycerol lipase). The HMP group showed significant upregulation of *PTPN22* expression levels at 2 weeks (*p* < 0.01). However, the expression levels of *DAGL-α* and *MAGL* were not significantly different between Con and HMP groups. Especially for the HP group, the ECS-related mRNA expression levels were downregulated progressively (2 weeks vs. 5 weeks) with the exception of *MAGL*. Overall, our results showed that multi-functional hydrogel coating could affect neuroprotective gene alteration including downregulation of neuroinflammation (*TNF-α, IL-1β*), upregulation of neurotrophic factors (*BDNF*, *Gfra1*), axonal maintenance and Schwann cell activation (*IL-6*, *Sox 10*, *S100B*, *P75* ^*NTR*^, *GAP43*), and myelination (*MBP*, *MPZ*, *NrCAM*, *NE-FL*). Such changes in gene expression may be a possible mechanism to explain the long-term biocompatibility enhancement of multi-functional hydrogel coatings. Consistent with these changes, CB1 could be associated with peripheral nerve specific-biological responses to neural electrodes such as Schwann cell activation and myelination.

### Immunofluorescence Staining

**Figure 6:**
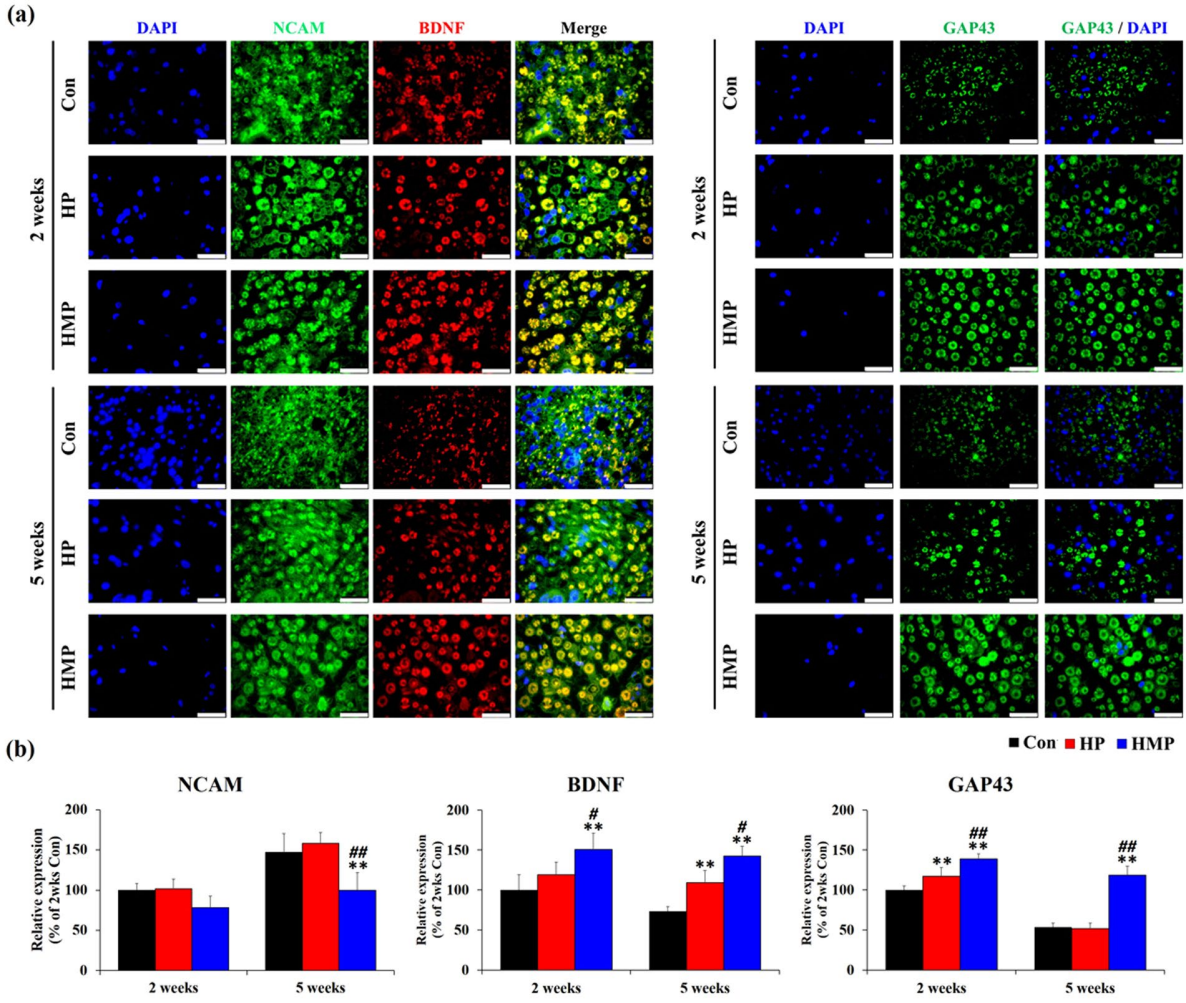
Immunofluorescence staining for NCAM, BDNF, and GAP43 in the sciatic nerves at 2 and 5 weeks post-implantation. (**a**) Immunofluorescence with antibodies to NCAM, GAP43 (green) and BDNF (red). Scale bar = 25 μm (**b**) Statistical analysis of immunofluorescence staining (n = 3 per each group and each time point). Results are expressed as mean ± SD. ^*^Compared to Con, ^#^Compared to HP, ^*, #^ *p* < 0.05, ^**, ##^ *p* < 0.01; One-Way ANOVA analysis.

Protein expression of three genes (NCAM, BDNF and GAP43) that were significantly reduced or increased at 5 weeks in the HMP groups were confirmed by immunofluorescence staining. Our experimental results showed that the expression patterns of BDNF (red) and GAP43 (green) were mainly related to axonal integrity (Fig. 6a). In the HMP group, BDNF and GAP43 positive axons were increased during experimental period compared to the Con and HP groups. Interestingly, NCAM (green) protein was expressed not only in the axonal structure, but also in the peri-neural tissues of the experimental groups. As a result, expression of NCAM protein was higher in Con and HP groups than in HMP group at 5 weeks.

Quantitative analysis revealed that the number of BDNF positive axons and GAP43 positive axons in the Con and HP groups decreased over time (Fig. 6b). However, the HMP group showed relatively constant BDNF and GAP43 positive axons and was significantly higher than the Con and HP groups (*p* < 0.05). On the other hands, NCAM expression was higher in Con and HP group than in HMP group. The Con and HP groups showed increased NCAM immunoreactivity in the peri-neural tissues from 2 to 5 weeks, while the HMP group remained relatively constant over time. These differences of NCAM expression were observed to be statistically significant at 5 weeks (*p* < *0.01*). Overall, these results indicated that the use of multi-functional hydrogel coating could affect biocompatibility through up-regulation of axonal regeneration-related gene and protein expressions.

**Figure 7:**
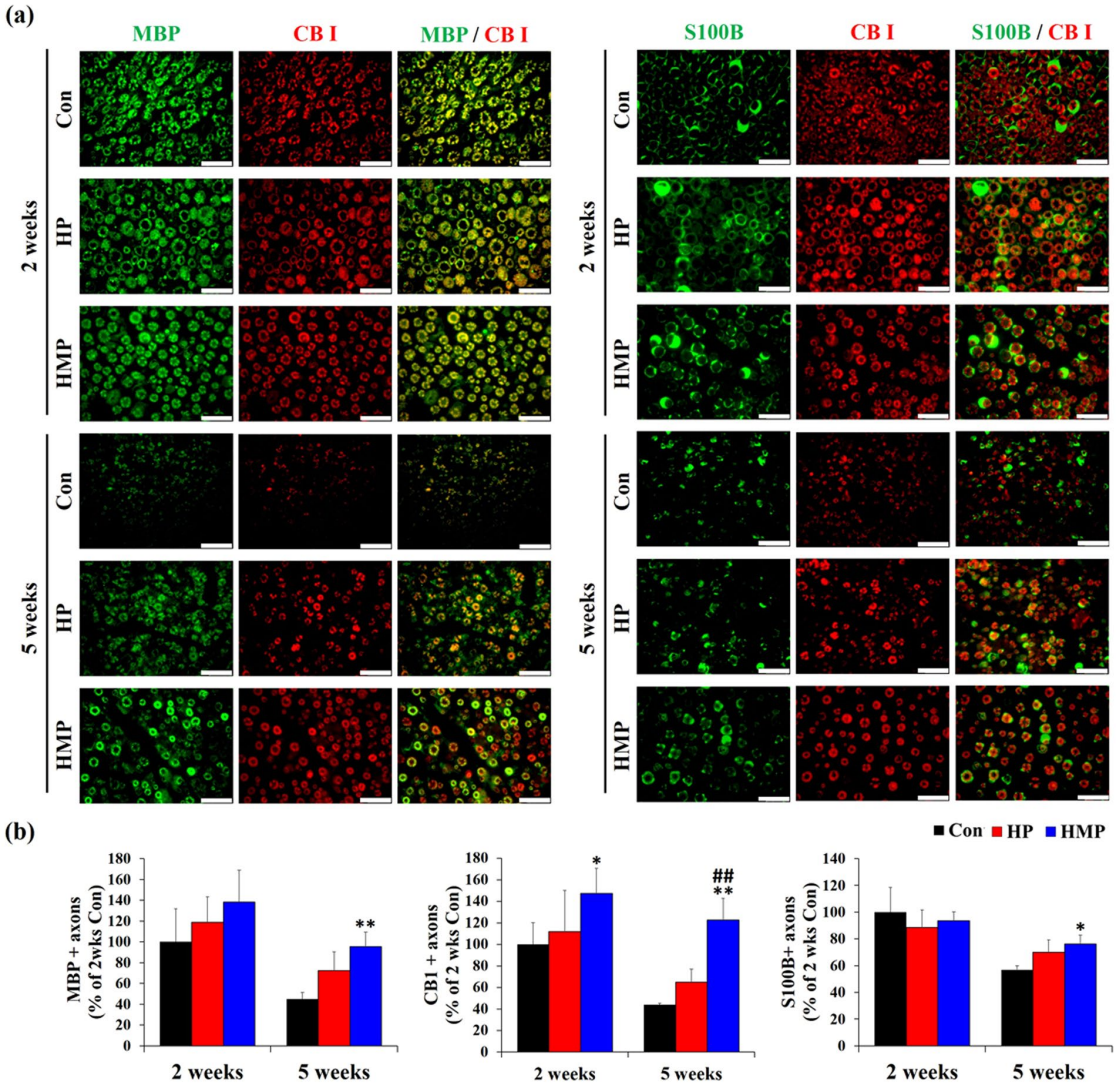
Double immunofluorescence staining for MBP, CB I, and S100B in the sciatic nerves at 2 and 5 weeks post-implantation. (**a**) Double immunofluorescence with antibodies to MBP, S100B (green) and CB I (red). Scale bar = 25 μm (**b**) Statistical analysis of double immunofluorescence staining (n = 3 per each group and each time point). Results are expressed as mean ± SD. *Compared to Con, ^#^Compared to HP, ^*, #^ *p* < 0.05, ^**, ##^ *p* < 0.01; One-Way ANOVA analysis.

We also confirmed the relationship between CB1 receptors and axonal maintenance using double immunofluorescence staining for S100B, CB1, and MBP proteins. Previous studies have reported that a wide range of dorsal root ganglion (DRG) neurons and axon fibers such as myelinated A-fiber cells and unmyelinated C-fiber cells expressed CB1 receptors in both normal and pathological conditions^23^. Microscopic examinations revealed that most of the axon fibers were double immune-reactive for MBP (green) and CB1 (red) at 2 weeks in all the experimental groups (Fig. 7a). Similar to the HE and LFB-CEV staining results, the number of MBP/CBI and S100B/CB1 double positive axons was decreased in the Con and HP groups at 5 weeks post-implantation. Only the HMP group maintained a relatively constant number of Schwann cells at 5 weeks. Furthermore, the HMP group showed an increased number of CB1/MBP double positive axon fibers (myelinated fiber) and CB1+/MBP- axon fibers (unmyelinated fiber) than the Con and HP groups at 5 weeks.

Quantitative analysis revealed that the number of axons positive for MBP and S100B positive Schwann cells decreased in most of the experimental groups (Fig. 7b). However, the degree of the decrease was relatively small in HMP group, and the number of MBP and/or S100B-positive axons was greater than that of the Con group (*p* < 0.05). Interestingly, the number of CB1 positive axons was similar to that of MBP and S100B positive axons in Con and HP groups. However, the HMP group showed a relatively constant number of CB1 positive axons over time (2 weeks vs. 5 weeks, *p* > 0.05), and the number of CB1 positive axons was much higher than that of Con and HP (*p* < 0.01). Along with gene expression analysis, these immunofluorescence results suggest that CB1 could be specifically associated with host biological response during the tissue remodeling step by Schwann cell activation and myelination. In this respect, the application of HMP to the electrodes has affected the recording function and structural maintenance of peripheral nerves mediated by upregulation of neuroprotective genes and by the increase of CB1-positive myelinated/unmyelinated axons.

## Discussion

Over the past several decades, research has focused on obtaining high-quality neural signals in long-term implantation through anti-biofouling material coatings, conductive material coatings, and modulation of host inflammatory responses. As with these efforts, we previously reported multi-functional hydrogel coated electrode which had advantages of electrochemical properties and anti-inflammatory drug (CsA) loading capacity^20^. In that report, the multi-functional hydrogel coating exhibited increased *in vitro* electrical conductivity and initial biocompatibility due to the presence of electrodeposited PEDOT:PSS layer, microwell pattern, and CsA-loaded microsphere. As part of further research, in this article, we focused on the *in vivo* function of the electrode and the biological response of the implanted host when applying a multi-functional hydrogel. Through this, we have analyzed the underlying biocompatible mechanisms of multi-functional hydrogel coating and explored the expression changes of the endocannabinoid system associated therewith.

Assessment of the host’s biological response to the biomaterial, especially in the case of long-term implantation, is one of the most important factors in successful *in vivo* function and ongoing monitoring. The host’s biological responses to cuff electrodes are regulated in a complex manner, and the response lasts up to the electrode placed around the nerve^11,12^. The host’s biological responses to the implanted devices initiated by the surface biofilm coating (protein absorption due to Vroman effect), acute inflammation (segmented neutrophil infiltration), foreign body response (granulation tissue formation, fibrous scar formation), and finally led to target tissue remodeling (cell death, gene expression alteration, regeneration, phenotype change)^11,12^. It is important to analyze biological events at the implant-tissue interface for a better understanding of successful/failed implants and for advances in technology for clinical applications.

Our *in vivo* electrode function analysis results showed that peak-to-peak amplitudes of electrically evoked ENG were increased in the surface-modified group (HP and HMP) at 2 weeks. These results could be influenced by the stable charge transfer properties of the PEDOT:PSS coating. Several previous studies have reported improved communication between nerve tissue and electrodes by applying a conductive polymer-hydrogel composite to the electrodes^24–27^. We have also previously reported the beneficial effects of PEDOT: PSS on charge transport properties, suggesting that electron-polymerized PEDOT:PSS on the electrode provides a more stable charge transfer than the bare electrode^27^. Thus, the PEDOT:PSS layers of the HP and HMP group exhibited improved charge transfer properties, finally leading to high-quality electrical signals.

However, the amplitude of both evoked ENG and afferent ENG in the Con and HP groups decreased over the course of 2 to 5 weeks. Most of the axonal maintenance factors such as epi/endoneurial fibrosis, axonal densities, distribution of axon diameter, and a number of TUNEL-positive apoptotic cells were gradually deteriorated in the Con and HP groups. It has been reported that modifying the surface of the electrode with only a non-fouling coating is not sufficient to reduce inflammatory cell recruitment or apoptosis^28^. In our results, surface modification with PEG hydrogel coating alone was not sufficient for axon survival and remodeling in chronic implantation.

Thus, drug-loaded multi-functional surface modification should be provided to maintain the high performance of the electrode itself and to maintain the axon structure during long-term management. Previous studies have shown that coating an anti-inflammatory agent such as dexamethasone could improve the tissue response around the implanted neural electrodes^29,30^. According to our results, the HMP group exhibited enhanced recording properties that relatively constant evoked ENG was observed during the experiment. In addition to improved electro-recording capabilities, the HMP group exhibited minimized fibrous tissue infiltration, reduced myelin degradation, and improved axonal cell survival. These results are likely to be based on the neuroprotective properties of CsA loaded on multifunctional hydrogels. CsA has been reported to have direct/indirect neuroprotective properties in neuropathies, such as neuronal progenitor cell activity and mitochondrial dysfunction associated ischemic brain injury^31,32^. In this respect, the results of morphological and histochemical results of the HMP group could be affected by a sufficient amount of CsA loading. Thus, it has been demonstrated that the CsA-loaded MS/PEG hydrogel coating could provide CsA on the electrode surface in an amount sufficient to provide neuroprotection properties over time as well as reduction of inflammation.

Our gene expression analysis results also revealed that the multi-functional hydrogel-coated electrode could alter the host’s biological response. Gutowski recently reported that surface-modified neural electrodes increased neuronal survival through decreased inflammatory cytokine release, and increased expression of *IL-6* and ciliary neurotrophic factor (*CNTF*)^33^. In our results, 20 genes were analyzed, and 12 neuroprotective genes showed significant differences between groups at 5 weeks. Using multifunctional hydrogels on the use the electrode, we confirmed that 12 genes (*BDNF*, *Gfra1*, *IL-6*, *Sox 10*, *S100B*, *P75* ^*NTR*^, *GAP43*, *MBP*, *MPZ*, *NrCAM*, *NE-FL*, *CB1*) were upregulated at 5 weeks of implantation. In addition, immunofluorescence staining revealed that protein expression of BDNF and GAP43 increased only in the HMP groups at 5 weeks post-implantation. These genes and proteins are linked to improved biocompatibility and neuroprotective properties in implanted hosts, which are essential for long-term maintenance. For example, although *IL-6* is traditionally thought of as a pro-inflammatory cytokine, there have been a number of studies that have been shown to play a role in Schwann cell myelination, cell-survival, and anti-apoptosis in the nervous system^33,34^. Also, similar to our results, neural adhesion and neuroskeletal molecules such as *NrCAM*, and *NE-FL* could be differentially regulated by axonal myelination (upregulation of *NrCAM* and *NE-FL*)^35–37^. Compared to the HMP group, NCAM gene and protein expression levels were significantly up-regulated in the Con and HP groups at 5 weeks. In general, NCAM is known to mediates intercellular adhesion and signaling in the nervous system^38^. However, it has been reported that NCAM is also expressed in non-neural tissues such the interstitial cells and portal fibroblast during regeneration^39,40^. Under normal circumstances, NCAM-positive stromal cells are very rare, but increase under certain pathological conditions such as early phase of interstitial fibrosis. Similar to the previous studies, our immunofluorescence results showed that the NCAM protein was expressed in the peri-neural tissues, especially in the Con and HP groups. Taken together, expression levels of neuroprotective genes and proteins could be altered by multi-functional hydrogel coatings, and these modulations can be essential for successful long-term implantation.

Interestingly, the surface-modified groups (HP, HMP) showed downregulation of the *CB2* gene expression consistent with *TNF-α* and *IL-1β*. Similar to our results, CB2 receptor expression is closely related to immune response, including a variety of neuropathies that exhibit a hyperinflammatory component^41^. Our results suggest that the analysis of CB2 expression profile might be used as a useful indicator of inflammation in biocompatibility studies of the peripheral nervous system.

Furthermore, gene expression analysis and double immunofluorescence showed the possibility of involvement of ECS in neuroprotection and peripheral nerves regeneration during cuff electrode implantation. It is well known that CB1 was detected in dorsal root ganglion (DRG) neurons and that CB1 is localized in both large-diameter non-nociceptive and small-diameter nociceptive neurons^17,18^. Interestingly, in our results, the gene expression level of CB1 was significantly increased in the HMP group only at 5 weeks in a pattern similar to the neurotrophic factors, Schwann cell activation, and myelination-related genes. Double immunofluorescence also revealed that both CB1-positive myelinated (CB1^+^/MBP^+^) and unmyelinated (CB1^+^/MBP^−^) axon fibers increased only in the HMP group at 5 weeks. Taken together, these results demonstrate the possible evidence for the role of CB1 receptors in neuroprotective properties and axonal phenotype change upon surface modification by multi-functional hydrogel coating. Indeed, the association of CB1 with a nervous system has been previously reported. Wolf *et al*. reported that CB1 affects the adult neurogenesis, such as proliferation, survival, and maturation of neural progenitor cells^42^. They concluded that CB1 expression could be reversed between proliferation (downregulated) and differentiation (upregulated) of neuronal cells. Other research groups also have shown that differentiation and myelination processes of oligodendrocyte, one of the nerve cells of central nervous system, are closely linked to the activation of cannabinoids, CB1 or CB2 through the mTOR and Akt signaling pathways^19^. In this regard, the increase in the numbers of CB1-positive axons in the HMP group may be one of the representative features of the tissue remodeling phase of the peripheral nervous system. Taken together, our results suggest that ECS can play a role in peripheral nerves and CB1 can be used as an index of phenotypic changes in peripheral nerve damage and regeneration.

## Methods

### Preparation of a multi-functional nerve cuff electrode

The process used to fabricate the nerve cuff electrode was described in detail in our previous reports^20,43^. The PEG hydrogel-coated group (HP, HMP) was surface modified to improve hydrogel adhesion using N-(3-Aminopropyl) methacrylamide hydrochloride (APMA) and tributylamine. Additional surface modification process was performed in the HMP group such that a microwell patterned electrode was made using positive photoresist (AZ 9260, AZ Electronic Materials, NJ, USA) and reactive ion etching (RIE) processes.

The CsA-loaded PLGA microsphere was prepared by the oil-in-water (O/W) emulsion/solvent evaporation method as previously described^44,45^. After microsphere preparation, the PEG and CsA loaded microsphere composite hydrogels were mixed and coated on the surface-modified PI-based cuff electrode by UV-induced chemical crosslinking (final concentration of CsA loading: 3 mg in 90 μl) (Fig. 1b,c). In addition, the PEDOT:PSS polymer was electro-polymerized on four Pt line electrode sites. For the electro-polymerization of PEDOT:PSS, the working electrode was connected to the nerve cuff electrode through an external connector. An Ag/AgCl reference electrode and Pt wire as a counter electrode were used. A current density of 8 μA/mm^2^ was applied to each line electrode site for 300 s.

### Surgical procedures

Eighteen 10-week-old male Sprague-Dawley (SD) rats, average weighing 350 g were purchased from Young Bio (Seongnam, Gyeonggi-do, Korea). All the animal experiments were approved by the Institutional Animal Care and Use Committee of Konkuk University (KU16029), and the methods were performed in accordance with the relevant guidelines and regulations. All the electrodes for the *in vivo* study were sterilized using a UV-C germicidal lamp equipped laminar-flow hood (UV output of 19.8 W, Sankyo Denki, Japan) for about 2 hours.

General anesthesia was induced using xylazine HCl (Bayer Korea, Gyeonggi-do, Korea) and maintained with 1.5–2% isoflurane (Hana Pharm, Gyeonggi-do, Korea) with 100% O2 during the surgical procedures. A mid-lateral skin incision was made a parallel to the femur, and a pair of electrodes was carefully implanted in the sciatic nerve. The gap between the stimulating and recording cuff electrodes was 5 mm. All the leads were routed subcutaneously to a pin connector for the continuous acquisition of data, and the connector was sealed with dental cement (VertexTM Self Curing, Vertex Dental, Soesterberg, Netherlands).

### In vivo electrical stimulation and recording

Electrically evoked compound nerve signals were recorded once a week until the end point of the experiments (n = 3 per group and time point). Biphasic periodic electrical stimulation (0.1 ms/phase, 300 μA peak current) was applied by a stimulating electrode, which was connected to pulse stimulator (Isolated Pulse Stimulator, Model 2100, A-M Systems, Sequim, WA, USA). The evoked compound nerve signal from the sciatic nerve was recorded by the implanted recording cuff electrodes. The recording cuff electrode was connected to a differential amplifier (Differential AC amplifier, Model 1700, A-M Systems) and the amplified neural signals were collected by a data acquisition (DAQ) device (NI USB-6356, National Instruments, Seoul, Korea). Next, the collected signals were processed using the LabVIEW software (National Instruments, Seoul, Korea) and displayed on a laptop computer. When a nerve is stimulated by the electrodes, an action potential is transmitted along the axon, enabling the nerve cells to communicate and activate a deliberate plantar movement (muscle contraction).

### Histological and histochemical analysis

The rats were sacrificed two and five weeks after implantation (n = 3 per each group) using CO^2^. The implanted cuff electrodes and the surrounding tissue were collected immediately and evaluated by histopathological and gene expression analysis. For the histopathological examination, the sciatic nerve was fixed with 10% neutral buffered formalin (BBC Biochemical, WA, USA), processed using the standard method, and embedded in paraffin. The cross sections (4 μm in thickness) of the sciatic nerves were stained with hematoxylin and eosin (H&E, BBC Biochemical) to analyze the axonal structure and the fibrous tissue infiltration around the nerve. Masson’s trichrome staining (BBC Biochemical) was performed to analyze intra-neural fibrous tissue infiltration. Myelin contents among the experimental groups were also assessed by the Luxol fast blue-cresyl etch violet staining (LFB-CEV, American MasterTech, CA, USA). Quantitative data including epineural fibrous tissue thickness and LFB-CEV positive myelinated axons were measured using the Leica Application Suite software (Leica Microsystems, Wetzlar, Germany). The number of axons was measured at 1000 × magnification in at least five different microscopic areas for each sample. Axonal density was calculated by dividing the average number of LFB-CEV positive myelinated axons by the microscopic field.

### Immunofluorescence staining

Serial formalin-fixed tissue sections were used for double immunofluorescence staining. Briefly, the sections were deparaffinized and underwent heat-induced antigen retrieval using a combination of citric acid (Sigma-Aldrich, MO, USA) and trisodium citrate (Sigma-Aldrich) to liberate the cross-linked epitopes. The sections were then incubated in blocking solution (3% bovine serum albumin, Sigma) for 30 min. The primary antibodies used were the monoclonal mouse anti-NCAM antibody (Abcam, 1:100), polyclonal rabbit anti-BDNF antibody (FineTest, Wuhan, China, 1:100), monoclonal mouse anti-GAP43 antibody (Santa Cruz Biotechnology, CA, USA, 1:100), monoclonal mouse anti-MBP antibody (Santa Cruz Biotechnology, 1:100), polyclonal rabbit anti-CB I (Abcam, Cambridge, UK, 1:200), and monoclonal mouse anti-S100B (Abcam, 1:100). After washing with 1X PBS for 3 × 5 min, they were incubated with a combination of FITC-conjugated goat anti-mouse and Texas Red-conjugated goat anti-rabbit secondary antibodies (Santa Cruz Biotechnology, 1:100) for 30 min. The sections were fixed with fluoromount (Vector Laboratories, CA, USA) and analyzed under a fluorescence microscope (Leica Microsystems).

### Apoptosis analysis

TUNEL staining was performed according to the manufacturer’s instructions (In Situ Cell Death Detection Kit, POD [Roche Diagnostics, Mannheim, Germany]). After the heat-induced antigen retrieval, the sections were incubated in the dark with the TdT enzyme and fluorochrome mixture for 1 h at 37 °C. After washing with 1X PBS for 3 × 5 min, the TdT-labeled sections were incubated with convertor POD for 30 min and then visualized using Vector SG (Vector Laboratories). The sections were counterstained with a nuclear fast solution.

### Real-time PCR analysis

Total RNA from the electrode implantation site (n = 3 per each group and time point) was isolated using Qiazol lysis reagent (Qiagen, CA, USA) according to the manufacturer’s instructions. One microgram of total RNA was transcribed into cDNA with a QuantiTect Reverse Transcription Kit (Qiagen). The primers of the measured mRNA genes are listed in Table 1. Real-time PCR was analyzed using a Rotor-Gene SYBR Green PCR Kit (Qiagen). The real-time PCR amplifications were conducted for 5 s at 95 °C and 10 s at 60 °C for 45 cycles after the initial denaturation step of 5 min at 95 °C. All the mRNA levels were normalized to the values of reference genes, and the results were expressed as fold changes of the threshold cycle (Ct) value relative to mean of the 2 weeks controls using the 2−ΔΔCt method^46^. The reference genes were validated that average expression of glyceraldehyde 3-phosphate dehydrogenase (GAPDH), 18 s ribosomal RNA (18 s rRNA), beta actin, and hypoxanthine phosphoribosyltransferase 1 (HPRT1) were used for the normalization of quantitative expression data.

### Statistical analysis

All the values were presented as mean ± standard deviation (SD). Statistical analysis was performed using Instat 3 (GraphPad Software, CA, USA) or SPSS Statistics software (version 19.0; IBM, Chicago, IL, USA). Multiple comparisons were analyzed using one-way analysis of variance (ANOVA) and a Bonferroni post-hoc paired comparison test. Histograms were compared using two-sample Kolmogorov–Smirnov test. Values of *p* < 0.05 and *p* < 0.01 were considered to be statistically significant.
